# Self-reported traumatic brain injury in a sample of impulsive violent offenders: neuropsychiatric correlates and possible “dose effects”

**DOI:** 10.3389/fpsyg.2023.1243655

**Published:** 2023-09-12

**Authors:** Vasudeva Murthy Challakere Ramaswamy, Tony Butler, Bianca Ton, Kay Wilhelm, Philip B. Mitchell, Lee Knight, David Greenberg, Andrew Ellis, Stephen Allnutt, Jocelyn Jones, Val Gebski, Vaughan Carr, Rodney J. Scott, Peter William Schofield

**Affiliations:** ^1^School of Medicine and Public Health, University of Newcastle, Newcastle, NSW, Australia; ^2^University of New South Wales, Sydney, NSW, Australia; ^3^Justice Health and Forensic Mental Health Network, Matraville, NSW, Australia; ^4^National Drug Research Institute, Curtin University, Perth, WA, Australia; ^5^NHMRC Clinical Trials Centre, University of Sydney, Camperdown, NSW, Australia; ^6^Department of Psychiatry, Monash University, Clayton, VIC, Australia; ^7^Neuropsychiatry Service, Hunter New England Mental Health, Newcastle, NSW, Australia

**Keywords:** traumatic brain injury, offending potential, impulsivity, orbitofrontal cortex (OFC), violent behavior

## Abstract

**Background:**

Traumatic brain injury (TBI) is a major public health problem that may be associated with numerous behavioral problems, including impulsivity, aggression and violence. Rates of self-reported TBI are high within offender populations, but the extent to which TBI is causally implicated in causing illegal behavior is unclear. This study examined the psychological and functional correlates of histories of traumatic brain injury in a sample of impulsive violent offenders.

**Methods:**

Study participants, all men, had been recruited to participate in a randomized controlled trial of sertraline to reduce recidivism. Study entry criteria were an age of at least 18 years, a documented history of two or more violent offenses and a score of 70 or above on the Barratt Impulsiveness Scale. An extensive list of standardized questionnaires was administered to obtain information on previous TBI and other neuropsychiatric conditions or symptoms.

**Results:**

In the sample of 693 men, 66% were aged between 18 and 35 years old, and 55% gave a history of TBI (“TBI+”). Overall, 55% of study participants reported at least one TBI. High levels of neuropsychiatric symptomatology were reported. In 75% of TBI+ individuals, their most severe TBI (by self-report) was associated with loss of consciousness (LOC) < 30 min. Compared to TBI- (those without history of TBI) participants, TBI+ individuals were more impulsive (Eysenck Impulsivity), irritable, angry, and reported higher levels of assaultive behavior, depressive symptomology, alcohol use disorder, suicidal ideation, suicide attempts, and lower quality of life. Potential “dose effects” of TBI severity and frequency in terms of neuropsychiatric symptomatology were identified.

**Conclusion:**

Like other studies of offender populations, single and multiple TBIs were very common. The associations of TBI, TBI severity, and TBI frequency (i.e., TBI “burden”) with adverse neuropsychiatric phenomena suggest TBI contributes importantly to offender morbidity but the select nature of the sample and cross-sectional study design constrain the interpretation of these findings.

## 1. Introduction

Violence, defined as “the intentional, threatened or actual, use of physical force or power, against oneself, another person, or a group or community, that either results in, or has a high probability of resulting in, injury, death, psychological harm, maldevelopment or deprivation” accounts for many millions of deaths world-wide annually, and is a major public health problem (Krug et al., 2002; Abubakar et al., 2015).

A history of TBI has been identified as one of many risk factors for violence and, conversely, TBI might arise as a consequence of violent behavior (Morrell et al., 1998; Slaughter et al., 2003; Schofield et al., 2006a). TBI is common in offender populations and, among individuals who have committed violent crimes (Fazel et al., 2011), and significantly exceeds rates in the general, non-offender population (Schofield et al., 2014).

The extent to which TBI might operate as a risk factor for violence or criminality remains unclear. Longitudinal and linkage studies, where the temporality of TBI can be established (i.e., that it precedes offending) have suggested an up to a two-fold increase in the risk of offending outcomes, but such studies generally lack any proxy measure for pre TBI behavioral characteristics that might represent a confound (Shiroma et al., 2010; Elbogen et al., 2015; Schofield et al., 2015; Ray and Richardson, 2017).

Specifically, the trait of impulsivity or poor self-control, is typically established early in life, and might explain an apparent association of TBI with offending, given that this trait increases the risk of both outcomes (Romer, 2010; Kocka and Gagnon, 2014). Lack of high quality, or indeed any, index of TBI severity often represents a limitation in studies of TBI in offender populations. What data are available are generally based on self-report, in large non-selected samples of offenders the report of TBI impact is mild, findings that might serve to cast doubt, in those instances (i.e., of mild TBI), on a likely causal association of TBI with subsequent offending.

In the present study, we had the opportunity to examine self-reported data on past TBI, neuropsychiatric symptoms and conditions and indices of impulsivity in a sample of impulsive individuals who had previously been found guilty of violent offenses. These data comprise baseline information from a larger randomized controlled trial of sertraline designed to determine whether that medication might reduce repeat offending (Butler et al., 2021). We used these data to examine associations between the presence and severity of TBI and various neuropsychiatric characteristics, seeking to determine the extent to which, in this sample of violent offenders, those with past TBI differed from those without such a history and whether “dose effects” related to frequency or severity of TBI related to subjective neuropsychiatric phenomena.

## 2. Materials and methods

### 2.1. Subjects

Study participants were all men who met the criteria for randomization in the ReINVEST study (Butler et al., 2021). That study enrolled men who were at least 18 years old and had a prior conviction or were charged (with or without imprisonment) for 2 or more violent offenses (e.g., manslaughter, robbery, assault) and scored 70 or higher on the Barratt Impulsiveness Scale (Barratt et al., 1995) Other requirements were that participants were able to communicate in English, were medically fit and could give informed consent (Butler et al., 2021). Participants with active major mental illness (major depression or psychosis/schizophrenia) were deemed ineligible for participation.

### 2.2. Traumatic brain injury questionnaire

Participants were asked to complete the medical history questionnaire which included the following questions about past episodes of TBI; “Have you ever had a head injury where you passed out or had a ‘blackout’?” and “How often have you experienced a head injury?” Up to five separate episodes of traumatic brain injury were recorded and participants were asked to report the TBI in order of severity. The first reported TBI was the most severe. The traumatic brain injury questionnaire included the following questions: How long were you unconscious (blackout)? When did this happen? Additional information included whether they suffered a skull fracture or bleeding on the head or surgical procedure on the head?

If the participant suffered multiple TBIs, they were instructed to provide information related to their most severe form of TBI under the first episode of TBI, followed by other episodes of TBIs. The burden of TBI was determined in this study based on LOC, the number of TBIs and duration since the severe TBI.

### 2.3. Demographics data

Demographic data was collected relating to age, gender, ethnicity, marital status, children, education, employment, and accommodation.

### 2.4. Psychiatric measures

Baseline assessments included administration of the Barratt Impulsiveness Scale (BIS-11) and the Eysenck Impulsivity Questionnaire (EIQ) providing information on state and trait indices of aspects of impulsive behavior. The Anger Irritability and Aggression Questionnaire (AIAQ) was used to measure the subjective level of anger and aggression in recent weeks and the State Trait Anger Expression Inventory (STAXI-2) was used to measure the subjective level of anger in different situations. The Beck Depression Inventory-II (BDI-II) examined symptoms in the past week potentially indicative of a mood disorder. The Alcohol Use Disorders Identification Test (AUDIT) measured alcohol consumption in the 12 months prior to incarceration. AUDIT identified safe, harmful and hazardous levels of alcohol consumption. The Kessler Psychological Distress Scale (K-10) is a 10-item questionnaire that provides a global measure of distress based on questions about anxiety and depressive symptoms in the past 4 weeks. The International Personality Disorder Examination (IPDE) was used to categorize participants as having Impulsive Personality Disorder, Dissocial Personality Disorder and Borderline Personality Disorder. As a measure of reading ability, Wechsler Individual Achievement Test Score was collected. In addition, details of substance use and abuse were collected and psychiatric assessment recorded information on suicide attempts, self-harm and sexual abuse as part of the routine reception interview.

### 2.5. Social measures

The Duke Social Support Scale provided information on social interaction and the Quality-of-Life Short Form Questionnaire (SF-12) assessed health-related quality of life.

### 2.6. Statistical analysis

Descriptive statistics were used to describe the sample characteristics, presence of TBI, self-reported duration of any LOC, time of occurrence, the severity of the head injury, symptoms following head injury, and overall response distribution of the psychological and functional measures. Hosmer-Lemeshow goodness of fit test based on chi-square formulation was used to investigate the association of the presence of TBI with sample characteristics, personality disorder, psychiatric assessment, and substance abuse. This procedure was repeated on different study outcomes including duration of LOC, time of occurrence, and duration of LOC among participants who reported only one episode of TBI. Fisher’s exact test was used when the expected count assumption of the chi-square test was not achieved. Meanwhile, One-way ANOVA (equal variance not assumed) was performed to investigate the study outcome with continuous numerical covariates including demographic characteristics, and psychological and functional measures. Pairwise comparison test using the Bonferroni method was applied where one-way ANOVA is significant in more than 2 groups. All statistical procedures were two-sided and computed on R4.3.3. All results are interpreted at the 5% significance level.

## 3. Results

Data cleaning procedure was employed prior to data analysis. Out of 935 participants, 244 participants with incomplete data were removed. The 244 participants were missing mostly TBI history, AUDIT, AIAQ, STAXI-2, substance abuse, SF-12, Duke and demographic data. A total of 693 individuals with complete data were used for the analyses. Table 1 shows the socio-demographic characteristics of the sample of impulsive violent offenders. About 66% of the study participants were between 18 and 35 years old. The majority of them were of non-Aboriginal descent (85%), and 86% lived with their parents, partners or relatives at the time of recruitment. Half of them had never married and 41% had been unemployed for at least 6 months. On average, the number of different schools before dropping out was 4.42 (*SD* = 3.46). One third had left school without completing a high school qualification. Three quarters (75%) had been suspended from school and 40% had been expelled.

**Table 1:** Demographic data based with TBI and without TBI.

Variables	Overall (*n* = 693)	TBI (*n* = 384)	No TBI (*n* = 309)	
	N (%)	N (%)	N (%)	*p-*value^a^
**Age (years)**				0.149
18–35	460 (66%)	246 (64%)	214 (69%)	
36–55	220 (32%)	128 (33%)	92 (30%)	
>55	13 (1.9%)	10 (2.6%)	3 (1%)	
**Ethnicity**				0.230^b^
Not Aboriginal and or Torres Strait Islander	589 (85%)	321 (84%)	268 (87%)	
Aboriginal	94 (14%)	58 (15%)	36 (12%)	
Torres Strait Islander	8 (1.2%)	3 (0.8%)	5 (1.6%)	
Both Aboriginal and/or Torres Strait Islander	2 (0.3%)	2 (0.5%)	0 (0%)	
**Marital status**				0.185^b^
Single (never married)	365 (53%)	194 (51%)	171 (55%)	
Regular partner	170 (25%)	97 (25%)	73 (24%)	
Married	113 (16%)	72 (19%)	41 (13%)	
Separated	33 (4.8%)	14 (3.6%)	19 (6.1%)	
Divorced	11 (1.6%)	6 (1.6%)	5 (1.6%)	
Not answered	1 (0.1%)	1 (0.3%)	0 (0%)	
**Number of children, mean (SD)**	1.76 (2.00)	1.86 (2.14)	1.65 (1.82)	0.355^c^
**Age at leaving school, mean (SD)**	15.66 (1.61)	15.55 (1.48)	15.80 (1.75)	0.084^c^
**Number of schools changed before stopping from studying mean (SD)**	4.51 (4.85)	4.58 (5.73)	4.42 (3.46)	0.460^c^
**Number of times suspended from school, mean (SD)**	11.43 (18.20)	11.14 (17.47)	11.83 (19.19)	0.900^c^
**Number of times expelled from school, mean (SD)**	2.15 (2.80)	2.01 (2.60)	2.35 (3.07)	0.140^c^
**Education**				0.823^b^
Never attended school	4 (0.6%)	2 (0.5%)	2 (0.6%)	
Completed primary school only	14 (2.0%)	7 (1.8%)	7 (2.3%)	
Left school with no qualification	226 (33%)	128 (33%)	98 (32%)	
School certificate	251 (36%)	146 (38%)	105 (34%)	
HSC/VCE/Leaving certificate	72 (10%)	37 (9.6%)	35 (11%)	
College certificate/diploma	24 (3.5%)	14 (3.6%)	10 (3.2%)	
Technical or Trade qualification	86 (12%)	43 (11%)	43 (14%)	
Degree/tertiary education	13 (1.9%)	5 (1.3%)	8 (2.6%)	
Not answered	3 (0.4%)	2 (0.5%)	1 (0.3%)	
**Employment**				**0.027**
Unemployed for at least 6 months	286 (41%)	174 (45%)	112 (36%)	
Employed within last 6 months	405 (58%)	209 (54%)	196 (63%)	
Not answered	2 (0.3%)	1 (0.3%)	1 (0.3%)	
**Accommodation**				**0.006** ^**b**^
Live alone	92 (13%)	46 (12%)	46 (15%)	
Partner	188 (27%)	121 (32%)	67 (22%)	
Mother/father	193 (28%)	89 (23%)	104 (34%)	
Mother/father and partner	14 (2.0%)	6 (1.6%)	8 (2.6%)	
Other relatives	202 (29%)	120 (31%)	82 (27%)	
Not answered	4 (0.6%)	2 (0.5%)	2 (0.6%)	
Have you ever attended any special schools/classes?				0.71
No	452 (65%)	246 (64%)	206 (67%)	
Yes	237 (34%)	136 (35%)	101 (33%)	
Have you ever been expelled from a school?				0.515
No	410 (59%)	220 (57%)	190 (61%)	
Yes	280 (40%)	162 (42%)	118 (38%)	
Have you ever been suspended from a school?				**0.02**
No	167 (24%)	78 (20%)	89 (29%)	
Yes	522 (75%)	304 (79%)	218 (71%)	

^a^Hosmer-Lemeshow Goodness of fit test. ^b^Fisher’s exact test. ^c^One-way ANOVA (equal variance not assured). SD, standard deviation. Bold values indicate statistical significance at 0.05 level. All statistical procedures were computed on R4.3.3. Statistical tests are two-sided.

As indicated in Table 2, 55% of study participants reported at least one TBI. Table 2 shows that the majority of episodes of LOC after traumatic brain injury lasted only a brief moment (63%) and this trend was also seen in participants with single episode of TBI (TBI 1) (62%), two episodes of TBI (TBI 2) (65%) and three episodes of TBI (TBI 3) (74%). In 75% of all TBI+ individuals, the most severe TBI was associated with LOC < 30 min, consistent with mild TBI, about half of the TBIs occurred more than 10 years ago and 11% of them were associated with skull fractures. One fifth (20%) of participants reported “weakness” in some part of their body after TBI.

**Table 2:** TBI characteristics by traumatic brain injury.

Variables	Yes, for TBI (*n* = 384)	TBI 1 (*n* = 182)	TBI 2 (*n* = 81)	TBI 3 (*n* = 42)
	N (%)	N (%)	N (%)	N (%)
Time length of loss of consciousness				
Only a brief moment	243 (63%)	112 (62%)	53 (65%)	31 (74%)
More than 10 min	47 (12%)	24 (13%)	10 (12%)	2 (4.8%)
More than 30 min	32 (8.3%)	16 (8.8%)	4 (4.9%)	3 (7.1%)
More than 24 h	22 (5.7%)	12 (6.6%)	4 (4.9%)	2 (4.8%)
Do not Know	35 (9.1%)	16 (8.8%)	7 (8.6%)	4 (9.5%)
Missing	5 (1.3%)	2 (1.1%)	3 (3.7%)	0 (0.0%)
Recency of last TBI				
Within past week	1 (0.3%)	0 (0.0%)	1 (1.2%)	0 (0.0%)
Within past month	4 (1.0%)	3 (1.6%)	0 (0.0%)	0 (0.0%)
Within past 6 months	12 (3.1%)	6 (3.3%)	2 (2.5%)	2 (4.8%)
Less than 2 years	34 (8.9%)	20 (11%)	6 (7.4%)	1 (2.4%)
Less than 5 years	64 (17%)	32 (18%)	10 (12%)	9 (21%)
Less than 10 years	85 (22%)	41 (23%)	19 (23%)	10(24%)
Over 10 years ago	182 (47%)	80 (44%)	41 (51%)	20(48%)
Missing	2 (0.5%)	0 (0.0%)	2 (2.5%)	0 (0.0%)
Severity of head injury				
Skull fracture	44 (11%)	23 (13%)	10 (12%)	3 (7.1%)
Closed head injury with intracranial bleeding	18 (4.7%)	10 (5.5%)	2 (2.5%)	2 (4.8%)
Surgical intervention	9 (2.3%)	5 (2.7%)	2 (2.5%)	1 (2.4%)
Missing	313 (82%)	144 (79%)	67 (83%)	36 (86%)
Symptom following head injury				
Weakness in any part of body	77 (20%)	41 (23%)	15 (19%)	3 (7.1%)
Poor concentration	35 (9.1%)	16 (8.8%)	8 (9.9%)	4 (9.5%)
Memory loss	22 (5.7%)	8 (4.4%)	5 (6.2%)	4 (9.5%)
Problem finding the right words when speaking	8 (2.1%)	5 (2.7%)	0 (0.0%)	2 (4.8%)
Problem with coordination or balance	6 (1.6%)	2 (1.1%)	3 (3.7%)	0 (0.0%)
Personality change	3 (0.8%)	3 (1.6%)	0 (0.0%)	0 (0.0%)
Anxiety or depression	10 (2.6%)	3 (1.6%)	3 (3.7%)	1 (2.4%)
Others	3 (0.8%)	1 (0.5%)	1 (1.2%)	0 (0.0%)
Missing	220 (57%)	103 (57%)	46 (57%)	28 (67%)

All statistical procedures were computed on R4.3.3. Statistical tests are two-sided.

Univariate analysis showed significant associations between any TBI with LOC and employment, accommodation, and suspension from school. The number of TBI cases was significantly higher among those who had been unemployed in the last 6 months (45%, *p* = 0.027) and who lived with their partner (32%, *p* = 0.006) or with relatives (31%, *p* = 0.006) and they had had higher school suspensions (79%, *p* = 0.02) than those without TBI.

Table 3 shows the psychological and functional factors in relation to the presence or absence of any reported past history of TBI. Univariate analyses revealed that TBI was significantly associated with higher scores for impulsivity, irritability, anger lability, direct assault, depression score, anger expression-out, anger expression-in, alcohol use disorder, suicidal ideation, and suicidal attempt. TBI was also significantly associated with lower quality of life score in terms of physical health and mental health according to the SF-12. Overall substance abuse (former and current) was not significant but when all the individual drugs were analyzed, former Methyl enedioxy methamphetamine (MDMA) substance abuse was significantly higher in those with TBI (*p* = 0.037).

**Table 3:** Participant psychological and functional measures by with TBI and without TBI.

Variables	Overall (*n* = 693)	TBI (*n* = 384)	No TBI (*n* = 309)	
	Mean (SD)	Mean (SD)	Mean (SD)	*P-*value^a^
**Barratt impulsiveness scale**	85 (10)	85.73 (9.76)	85.06 (9.97)	0.340
**Eysenck impulsivity questionnaire**				
Impulsiveness	13.6 (3.7)	13.92 (3.48)	13.16 (3.95)	**0.015**
Venturesomeness	11.27 (3.07)	11.43 (3.01)	11.07 (3.14)	0.128
Empathy	11.5 (3.6)	11.58 (3.53)	11.38 (3.75)	0.516
**Anger, irritability, and assault questionnaire**				
Irritability	19 (6)	19.00 (5.82)	18.06 (6.32)	**0.016**
Anger lability	11.8 (4.9)	12.19 (4.90)	11.41 (4.86)	**0.038**
Direct assault	18 (7)	18.15 (6.95)	16.69 (6.72)	**0.002**
Verbal assault	17.0 (5.2)	17.30 (5.12)	16.64 (5.35)	0.062
Indirect assault	7.1 (3.6)	7.30 (3.63)	6.79 (3.49)	0.055
**Beck depression inventory**	11 (9)	11.74 (8.86)	10.13 (8.24)	**0.012**
**Kessler psychological distress scale**				
k10	15 (9)	15.36 (8.62)	14.39 (8.96)	0.081
**Duke social support scale**				
Duke score	24.2 (4.6)	24.12 (4.51)	24.24 (4.65)	0.714
**Quality of life short form questionnaire**				
Physical component	53 (7)	52.53 (7.92)	53.96 (6.62)	**0.024**
Mental component	41 (12)	39.82 (12.45)	42.09 (12.22)	**0.016**
**State–trait anger expression inventory**				
State anger	16.9 (4.5)	17.00 (4.86)	16.72 (4.12)	0.143
Trait anger	25 (7)	25.19 (6.42)	24.50 (6.78)	0.098
Anger expression-out	20.6 (5.0)	21.03 (4.90)	20.06 (5.03)	**0.003**
Anger expression-in	19.2 (4.3)	19.60 (4.12)	18.77 (4.52)	**0.009**
Anger control-out	17.4 (4.7)	17.28 (4.55)	17.64 (4.87)	0.323
Anger control-in	18.4 (4.9)	18.22 (4.80)	18.52 (5.09)	0.367
Anger expression-index	52 (14)	53.13 (12.88)	50.66 (14.69)	**0.007**
**Alcohol use disorders identification test**	11 (9)	11.83 (9.01)	9.90 (8.14)	**0.007**
**International personality disorder examination**				
Borderline	323 (47%)	186 (48%)	137 (44%)	0.282^b^
Impulsive	502 (72%)	278 (72%)	224 (72%)	0.978^b^
Dissocial	320 (46%)	188 (49%)	132 (43%)	0.101^b^
**Wechsler individual achievement test score, mean (SD)**	116 (16)	116.18 (16.66)	116.56 (15.04)	0.941^c^
**Self-harm/Suicide and past sexual abuse**				
Suicidal ideation	351 (51%)	217 (57%)	134 (43%)	**<0.001** ^**b**^
Suicidal attempt	172 (25%)	109 (28%)	63 (20%)	**0.015** ^**b**^
Self-harm or injury	169 (24%)	101 (26%)	68 (22%)	0.191^b^
Childhood sexual abuse	277 (40%)	162 (42%)	115 (37%)	0.184^b^
**Substance abuse (current)**				0.103
Yes	570 (82%)	324 (84%)	246 (80%)	
No	123 (18%)	60 (16%)	63 (20%)	
**Substance abuse (former)**				0.113
Yes	578 (83%)	328 (85%)	250 (81%)	
No	115 (17%)	56 (15%)	59 (19%)	
**MDMA**				**0.037**
Current	41 (5.9%)	19 (4.9%)	22 (7.1%)	
Former	244 (35%)	152(40%)	92 (30%)	
Never used	4(0.6%)	2 (0.5%)	2 (0.5%)	

^a^One-way ANOVA (equal variance not assured; Bonferroni post Hoc test where significant). ^b^Hosmer-Lemeshow Goodness of fit test. SD standard deviation. Bold values indicate statistical significance at 0.05 level. All statistical procedures were computed on R4.3.3. Statistical tests are two-sided. ^c^One-way ANOVA (equal variance not assured).

In Table 4, we compared groups defined by the reported severity (duration of LOC) among those who reported only a single TBI. The only significant differences were that those with LOC >30 min (mean = 50.76) scored lower on the quality-of-life physical health score and on the Duke Social support scale than those with LOC <30 min (Table 4).

**Table 4:** Participant psychological and functional measures by severity of brain injury based on people LOC (minutes) and people who had only one episode of TBI.

Variables	Brief LOC (*n* = 112)	>10 M (*n* = 24)	>30 M (*n* = 28)	
	Mean (SD)	Mean (SD)	Mean (SD)	*P-*value^a^
**Barratt impulsiveness scale**	86.46 (10.21)	86.42 (9.96)	86.54 (10.49)	0.98
**Eysenck impulsivity questionnaire**				
Impulsiveness	13.85 (3.61)	14.42 (3.11)	13.00 (3.68)	0.279
Venturesomeness	11.35 (2.57)	11.62 (2.50)	10.68 (3.17)	0.574
Empathy	11.40 (3.39)	11.33 (3.28)	11.18 (3.68)	0.972
**Anger, irritability, and assault questionnaire**				
Irritability	19.12 (5.48)	18.42 (7.82)	18.68 (6.49)	0.994
Anger lability	12.37 (4.77)	12.50 (4.03)	12.14 (4.48)	0.877
Direct assault	18.60 (7.20)	18.54 (5.79)	16.11 (7.30)	0.221
Verbal assault	18.08 (4.93)	17.46 (5.23)	17.07 (5.67)	0.682
Indirect assault	7.84 (3.84)	8.08 (3.49)	6.68 (4.01)	0.333
**Beck depression inventory**	10.34 (8.11)	9.88 (7.70)	12.89 (10.07)	0.404
**Kessler psychological distress scale**				
k10	14.25 (8.52)	11.50 (7.30)	15.32 (9.01)	0.286
**Duke social support scale**				
Duke score	25.14 (3.94)	26.42 (4.45)*	22.75 (4.41)*	**0.004**
**Quality of life short form questionnaire**				
Physical component	53.84 (5.94)	54.40 (8.14)*	50.76 (6.93)*	**0.021**
Mental component	41.99 (12.18)	43.24 (11.97)	40.33 (14.72)	0.811
**State–trait anger expression inventory**				
State anger	16.66 (4.01)	16.33 (3.32)	17.36 (4.60)	0.728
Trait anger	24.94 (6.46)	24.71 (6.97)	25.93 (6.29)	0.854
Anger expression-out	21.72 (5.13)	21.12 (5.32)	22.64 (4.65)	0.723
Anger expression-in	19.17 (3.62)	19.46 (5.01)	18.64 (3.92)	0.719
Anger control-out	17.44 (4.59)	17.75 (5.14)	16.07 (6.21)	0.215
Anger control-in	18.42 (4.67)	19.62 (6.08)	17.07 (5.50)	0.262
Anger expression-index	53.04 (12.97)	51.21 (15.90)	56.14 (14.16)	0.585
**Alcohol use disorders identification test**	10.78 (8.77)	10.62 (8.20)	9.93 (9.73)	0.64
**International personality disorder examination**				
Borderline	50 (45%)	10 (42%)	13 (46%)	0.941^b^
Impulsive	82 (73%)	17 (71%)	19 (68%)	0.845^b^
Dissocial	51 (46%)	8 (33%)	12 (43%)	0.548^b^
**Self-harm/Suicide and past sexual abuse**				
Suicidal ideation	51 (46%)	13 (54%)	17 (61%)	0.313^b^
Suicidal attempt	28 (25%)	7 (29%)	7 (25%)	0.911^b^
Self-harm or injury	25 (22%)	3 (12%)	8 (29%)	0.372^b^
Childhood sexual abuse	35(31%)	11 (46%)	15(54%)	0.059^b^
**Substance abuse (current)**				0.746
Yes	100 (89%)	22 (92%)	24 (86%)	
No	12 (11%)	2 (8.3%)	4 (14%)	
**Substance abuse (former)**				0.296
Yes	101 (90%)	19 (79%)	25 (89%)	
No	11 (9.8%)	5 (21%)	3 (11%)	

*Significant pairwise comparison (Bonferroni method). ^a^One-way ANOVA (equal variance not assured; Bonferroni post Hoc test where significant). ^b^Hosmer-Lemeshow Goodness of fit test. SD, standard deviation. Bold values indicate statistical significance at 0.05 level. All statistical procedures were computed on R4.3.3. Statistical tests are two-sided.

We then sought further evidence for possible “dose effects” of TBI by comparing groups defined by reported numbers of TBIs (1, 2, >2) (Table 5). Here, there were more differences. For the most part, the characteristics of those who reported only one TBI were similar to those reporting 2 TBIs, while those reporting >2 TBIs differed with respect to the less frequent categories in more ways. Thus, the number of TBIs was significantly, positively associated with impulsiveness (Barratt), level of depression, psychological distress, alcohol use disorder (AUDIT), dissocial personality, suicidal ideation and suicidal attempt, and inversely with social support and quality of life. Substance abuse overall (former and current) was not significant, but when analyzing all individual drugs, current cannabis abuse was significantly higher among those >2TBI (50%, *p* = 0.026).

**Table 5:** Participant psychological and functional measures by severity of head injury based on Number of TBI.

Variables	1 TBI (*n* = 182)	2 TBI (*n* = 81)	>2 TBI (*n* = 121)	
	Mean (SD)	Mean (SD)	Mean (SD)	*P-*value^a^
**Barratt impulsiveness scale**	86.04 (10.14)	82.81 (9.11)*	87.20 (9.26)*	**0.005**
**Eysenck impulsivity questionnaire**				
Impulsiveness	13.66 (3.67)	13.81 (3.05)	14.39 (3.44)	0.076
Venturesomeness	11.27 (2.69)	11.37 (3.13)	11.71 (3.38)	0.12
Empathy	11.38 (3.37)	12.02 (3.63)	11.59 (3.68)	0.279
**Anger, irritability, and assault questionnaire**				
Irritability	18.93 (6.05)	18.75 (5.72)	19.27 (5.57)	0.907
Anger lability	12.14 (4.63)	11.81 (5.13)	12.53 (5.17)	0.569
Direct assault	18.10 (6.98)	17.98 (6.75)	18.35 (7.09)	0.818
Verbal assault	17.64 (5.10)	17.17 (4.89)	16.87 (5.31)	0.43
Indirect assault	7.58 (3.79)	7.17 (3.14)	6.98 (3.68)	0.296
**Beck depression inventory**	10.84 (8.61)	10.53 (9.26)*	13.89 (8.63)*	**<0.001**
**Kessler psychological distress scale**				
k10	14.22(8.56)*	15.77 (9.34)	16.81 (8.03)*	**0.024**
**Duke social support scale**				
Duke score	24.73 (4.33)*	24.46 (4.26)	22.98 (4.76)*	**0.005**
**Quality of life short form questionnaire**				
Physical component	52.89 (7.00)	52.94 (7.87)	51.71 (9.16)	0.833
Mental component	41.79(12.55)*	39.57 (11.54)	37.01 (12.42)*	**0.005**
**State–trait anger expression inventory**				
State anger	16.81 (4.08)	17.11 (4.56)	17.22 (6.04)	0.713
Trait anger	25.10 (6.63)	25.09 (5.74)	25.40 (6.58)	0.815
Anger expression-out	21.52 (5.16)	20.38 (4.43)	20.73 (4.75)	0.08
Anger expression-in	19.10 (3.93)	20.01 (4.38)	20.08 (4.18)	0.099
Anger control-out	17.37 (4.84)	17.36 (4.24)	17.10 (4.34)	0.814
Anger control-in	18.37 (4.92)	18.64 (4.83)	17.72 (4.58)	0.432
Anger expression-index	52.88 (13.50)	52.40 (11.53)	53.99 (12.84)	0.573
**Alcohol use disorders identification test**	10.59 (9.06)*	12.17 (8.82)	13.45 (8.86)*	**0.011**
**International personality disorder examination**				
Borderline	78 (43%)	40 (49%)	68 (56%)	0.074^b^
Impulsive	131 (72%)	58 (72%)	89 (74%)	0.941^b^
Dissocial	78 (43%)*	40 (49%)	70 (58%)*	**0.038** ^**b**^
**Self-harm/Suicide and past sexual abuse**				
Suicidal ideation	90 (49%)*	49 (60%)	78 (64%)*	**0.026** ^**b**^
Suicidal attempt	46 (25%)*	18 (22%)	45 (37%)*	**0.030** ^**b**^
Self-harm or injury	42 (23%)	18 (22%)	41 (34%)	0.072^b^
Childhood sexual abuse	70 (38%)	32 (40%)	60 (50%)	0.136^b^
**Substance abuse (current)**				0.044
Yes	162 (89%)	63 (78%)	99 (82%)	
No	20 (11%)	18 (22%)	22 (18%)	
**Substance abuse (former)**				0.164
Yes	162 (89%)	67 (83%)	99 (82%)	
No	20 (11%)	14(17%)	22(18%)	
**Cannabis**				**0.026**
Current	89 (49%)	23 (28%)*	61 (50%)*	
Former	67 (37%)	40 (49%)	47 (39%)	
Never	1 (0.5%)	0 (0%)	1 (0.8%)	

*Significant pairwise comparison (Bonferroni method). ^a^One-way ANOVA (equal variance not assured; Bonferroni post Hoc test where significant). ^b^Hosmer-Lemeshow Goodness of fit test. SD, standard deviation. Bold values indicate statistical significance at 0.05 level. All statistical procedures were computed on R4.3.3. Statistical tests are two-sided.

We then compared TBI- participants with TBI+ participants who reported a history of a single mild TBI. In univariate analyses (Table 6), those with mild TBI had significantly higher scores than those with no TBI on scales of direct assault (mean = 18.60, *p* = 0.006), verbal assault (mean = 18.08, *p* = 0.008), indirect assault (mean = 7.84, *p* = 0.009), anger expression-out (mean = 21.72, *p* < 0.001), and anger expression-index (mean = 53.04, *p* = 0.043). Furthermore, relative to TBI-, those with a single mild TBI included a significantly higher proportion of current (89%, *p* = 0.022) and former (90%, *p* = 0.024) substance abusers.

**Table 6:** Participant psychological and functional measures by mild TBI and without TBI.

Variables	Overall (*n* = 421)	Mild TBI (*n* = 112)	No TBI (*n =* 309)	
	**Mean (SD)**	**Mean (SD)**	**Mean (SD)**	***P-*value** ^**a**^
**Barratt impulsiveness scale**	85 (10)	86.46 (10.21)	85.06 (9.97)	0.214
**Eysenck impulsivity questionnaire**				
Impulsiveness	13.3 (3.9)	13.85 (3.61)	13.16 (3.95)	0.123
Venturesomeness	11.14 (3.00)	11.35 (2.57)	11.07 (3.14)	0.743
Empathy	11.4 (3.7)	11.40 (3.39)	11.38 (3.75)	0.903
**Anger, irritability, and assault questionnaire**				
Irritability	18 (6)	19.12 (5.48)	18.06 (6.32)	0.05
Anger lability	11.7 (4.9)	12.37 (4.77)	11.41 (4.86)	0.11
Direct assault	17 (7)	18.60 (7.20)	16.69 (6.72)	**0.006**
Verbal assault	17.0 (5.3)	18.08 (4.93)	16.64 (5.35)	**0.008**
Indirect assault	7.1 (3.6)	7.84 (3.84)	6.79 (3.49)	**0.009**
**Beck depression inventory**	10 (8)	10.34 (8.11)	10.13 (8.24)	0.749
**Kessler psychological distress scale**				
k10	14 (9)	14.25 (8.52)	14.39 (8.96)	0.99
**Duke social support scale**				
Duke score	24.5 (4.5)	25.14 (3.94)	24.24 (4.65)	0.124
**Quality of life short form questionnaire**				
Physical component	54 (6)	53.84 (5.94)	53.96 (6.62)	0.582
Mental component	42 (12)	41.99 (12.18)	42.09 (12.22)	0.951
**State–trait anger expression inventory**				
State anger	16.7 (4.1)	16.66 (4.01)	16.72 (4.12)	0.328
Trait anger	25 (7)	24.94 (6.46)	24.50 (6.78)	0.402
Anger expression-out	20.5 (5.1)	21.72 (5.13)	20.06 (5.03)	**<0.001**
Anger expression-in	18.9 (4.3)	19.17 (3.62)	18.77 (4.52)	0.245
Anger control-out	17.6 (4.8)	17.44 (4.59)	17.64 (4.87)	0.759
Anger control-in	18.5 (5.0)	18.42 (4.67)	18.52 (5.09)	0.767
Anger expression-index	51 (14)	53.04 (12.97)	50.66 (14.69)	**0.043**
**Alcohol use disorders identification test**	10 (8)	10.78 (8.77)	9.90 (8.14)	0.407
**International personality disorder examination**				
Borderline	187 (44%)	50 (45%)	137 (44%)	0.955^b^
Impulsive	306 (73%)	82 (73%)	224 (72%)	0.883^b^
Dissocial	183 (43%)	51 (46%)	132 (43%)	0.606^b^
**Self-harm/Suicide and past sexual abuse**				
Suicidal ideation	185 (44%)	51 (46%)	134 (43%)	0.692^b^
Suicidal attempt	91 (22%)	28 (25%)	63 (20%)	0.310^b^
Self-harm or injury	93 (22%)	25 (22%)	68 (22%)	0.945^b^
Childhood sexual abuse	150 (36%)	35(31%)	115 (37%)	0.259^b^
**Substance abuse (current)**				**0.022** ^**b**^
Yes	346 (82%)	100 (89%)	246 (80%)	
No	75 (18%)	12 (11%)	63 (20%)	
**Substance Abuse (Former)**				**0.024** ^**b**^
Yes	351 (83%)	101 (90%)	250 (81%)	
No	70 (17%)	11 (9.8%)	59 (19%)	

^a^One-way ANOVA (equal variance not assured; Bonferroni post Hoc test where significant). ^b^Hosmer-Lemeshow Goodness of fit test. SD, standard deviation. Bold values indicate statistical significance at 0.05 level. All statistical procedures were computed on R4.3.3. Statistical tests are two-sided.

## 4. Discussion

In this sample of highly selected, impulsive and violent men, 55% reported a history of at least one TBI associated with some alteration of consciousness, a prevalence consistent with rates found in prisoner populations in the literature (Farrer and Hedges, 2011; Shiroma et al., 2012; Durand et al., 2017). Although the majority of TBIs in our sample were, on the basis of duration of self-reported LOC, mild, a considerable number were potentially associated with complications (skull fracture and “body weakness,” albeit transient). Further, in terms of the “dose” of TBI within this sample, 47% of those with any TBI reported only one, 21% reported two TBIs and the remaining 31% reported three or more TBIs. Again, this aligns with the results of other previous studies in prison/offender populations which indicate that a history of multiple TBIs is far from uncommon (Barnfield and Leathem, 1998; Slaughter et al., 2003; Schofield et al., 2006a,b).

TBI aside, neuropsychiatric conditions are highly prevalent in offender populations, and the current sample is no exception. Based upon the International Personality Disorder Examination, impulsive personality disorder was present in over 70% of the sample, likely a reflection of their offending history and the recruitment criteria requiring a threshold (high) score on the Barratt Impulsiveness Scale for inclusion. Borderline (47%) and dissocial (46%) personality disorders were also extremely common. Reflective of early life disadvantage, a history of sexual abuse was highly prevalent as were high rates of past suicidal ideation, self-harm or suicidal attempts. Such findings are particularly notable in light of the exclusionary criteria such that individuals with active major mental illness were not recruited into the ReINVEST study (Butler et al., 2021). High levels of anger and distress, and current and past substance abuse were other notable findings.

There is a growing literature relating to TBI within offender populations and the possible etiological/causal role of TBI for offending and the special needs/challenges that prisoners with a history of TBI might present. This is important as some of their behaviors (including impulsivity), may reflect neuropsychiatric sequelae of TBI. Cross sectional studies are inherently limited with respect to establishing causal relationships, but they may nevertheless yield interesting results of relevance to this issue. In a previous study by our group, we collected “control” data by telephone interview to supplement previously-collected data from recent prison entrants (Schofield et al., 2006b; Perkes et al., 2011). Past TBI history and some simple behavioral data, including questionnaire-derived estimates of impulsivity, were obtained from both samples. The control group (non-offending men) was purposefully recruited from the same regions of residence as the pre-incarceration residences of the offenders (also all male). Surprisingly, 82% of prisoners and 71.5% of community participants reported at least one past TBI of any severity (prevalence not significantly different) while levels of impulsivity were profoundly lower (*p* < 0.001) in the community non-offending sample (Perkes et al., 2011). When the history of any TBI with LOC was compared (64.5% of prisoners and 32.2%) the difference was significant but still far less so than the difference in impulsivity scores, which were comparably high among prisoners with and without past TBI history. Such findings speak to the complexity of the TBI-offending association (including the importance of severity of TBI when exploring its potential role in offending), the potential high relevance of impulsivity for offending and the relevance of socioeconomic status for TBI prevalence (i.e., given the very high rates in the control sample).

The present study shares certain features with our earlier (Perkes et al., 2011) study described above, but there are also important differences. The present offending sample was highly select (previously violent and impulsive) and, also by contrast with the earlier study, we did not recruit a separate control sample. Thus, for the present study, we were interested in the “burden” of neuropsychiatric morbidity and the association of these with self-reported TBIs in our sample who were at high risk of reoffending due to their impulsivity and past history of violent offending. While the present analyses do not directly contribute to the literature that seeks to clarify the extent to which TBI plays a role in the offending cycle, they do set the scene for reflections on the current status of that issue, in light of recent data from other studies, discussed below.

On the question of causation, the relevant studies that are most methodologically sound are longitudinal, often linkage, studies in which the temporal sequence of TBI exposure and the offending outcome can be more or less assured (Timonen et al., 2002; Fazel et al., 2011; Schofield et al., 2015). Typically, however, such studies lack data on TBI severity or any proxy for pre-injury behavioral characteristics. Both of these issues are of some relevance to the plausibility of TBI playing a causal role in the commission of an offense. Several large record linkage studies have adduced evidence suggesting that TBI constitutes a risk factor for offending, and a longitudinal birth cohort study in which adjustment was made for preinjury early behavior problems obtained similar findings (Timonen et al., 2002; Fazel et al., 2011; McKinlay et al., 2014; Schofield et al., 2015). However, studies in which a control cohort comprised individuals who sustained non-TBI injuries found no heightened risk for offending among those with TBI, relative to that control group (Kennedy et al., 2017; Bonow et al., 2019). That provocative finding raised the possibility that (preinjury) impulsivity or risk-taking behavior (such as might increase risk for *any* injury) might represent a confound. Seen in this light, the occurrence of a mild TBI might constitute a proxy for risk taking or impulsivity that represents the actual underlying risk factor for offending behavior. The plausibility of the hypothesis that mild TBI may be causally implicated in offending depends on the nature of the neuropsychiatric consequences of TBI that might be anticipated, either following a single event or when multiple instances occur.

It has been long recognized that multiple TBIs, even when classified as mild TBI, can have cumulative deleterious effects (Gronwall and Wrightson, 1975; Carlsson et al., 1987). There is increasing concern about repetitive TBI in contact sports athletes (Mayer et al., 2017) being associated with symptoms of depression, anxiety and cognitive impairment (Bailes et al., 2014) or, more controversially, chronic traumatic encephalopathy (Geddes et al., 1999; Omalu et al., 2005; McKee et al., 2009; Gavett et al., 2011; Russo et al., 2023), associated with symptoms of depression, impulsivity, aggression and increased suicidality (McKee et al., 2009, 2013). TBI sequelae such as post-traumatic stress disorder (PTSD), depression and generalized anxiety disorder are associated with overactivity of the underlying neural circuits and increased risk of impulsive aggression (Mattson, 2003). PTSD is a debilitating condition characterized by symptoms such as avoidance behavior, hyperarousal and intrusive memories but may also be associated with antisocial behaviors such as aggression, anger, impulsivity and suicidal tendencies (Dileo et al., 2008).

In our study, there were significant differences between groups defined by the number of reported TBIs (1, 2, >2), consistent with possible “dose effects” with respect to Beck Depression Inventory, psychological distress, quality of life, suicide ideation and suicidal attempts. The >2 TBIs category also had lower social supports, and were most impulsive of the three categories of TBI frequency without an obvious gradient of effect. That the >2 TBIs group reported greater substance abuse, including alcohol, may perhaps reflect consequences rather than causes of those exposures, recognizing that all of the above is necessarily speculative.

It has become increasingly apparent that a single mild TBI may be complicated by long-term physical, cognitive, and emotional consequences which may include an increase in aggression and impulsivity. When considering the effects of a single mild TBI, it is important to note that the definition encompasses a considerable spectrum of severity, e.g., from transient alterations in awareness to LOC lasting up to 30 min on one clinical measure (McKinlay et al., 2016; McInnes et al., 2017; Mosti and Coccaro, 2018).

Results from recent large cohort studies with pre-injury assessments have substantially contributed to our knowledge of complications by reporting actual change scores associated with the TBI, meaning that post TBI symptoms cannot be solely attributable to pre-injury characteristics. The Project on Human Development in Chicago Neighborhoods study involved a longitudinal assessment of 1,827 adolescents with serial assessments (Connolly and McCormick, 2019). Multivariate analyses were conducted to investigate the relationship between mild TBI and a range of important symptoms and, importantly, investigators were able to measure changes in key indices of neuropsychiatric functioning associated with a new mild TBI while adjusting for preexisting, previously measured, characteristics. Their data compellingly demonstrated increases in aggressive behavior, anxiety and depression and delinquency among those with a mild TBI (Connolly and McCormick, 2019). Studies of veterans exposed to mild TBI caused by blast injuries are also instructive and relevant. The context and unpredictable nature of blast injury in theaters of war is such that it is relatively implausible to invoke the victims’ own behavior as a risk for its occurrence. The frequency of the event, availability of relevant preinjury behavioral/psychometric data and availability of appropriate control data from uninjured victims has yielded abundant data, albeit recognizing that blast mild TBI may have its own unique characteristics and thus not constitute the perfect model for non-blast sustained mild TBI. In one recent study, indices of disinhibition were significantly elevated in veterans with blast induced mild TBI relative to their preinjury scores and to non-injured controls (Schindler et al., 2017).

In relation to the possible dose effects of a single TBI, we established categories of severity based upon the duration of LOC (Table 4) for those individuals in our study who reported one past TBI only. Here, significant differences, with the most severe group being associated with the most adverse circumstances, were reported with respect to the physical quality of life and social support only. When we compared individuals who had sustained a single mild TBI with the remaining sample of TBI- individuals (Table 6), there were significant differences with respect to levels of anger and substance use (in both instances more in the mild TBI group). The significance of those latter group associations is especially unclear as bidirectional causation is plausible (that is, anger and substance abuse might both lead to, or be consequences of, TBI).

It is well established that socio-demographic variables such as age, poverty and unemployment are influential in terms of an individual’s criminal risk (Krug et al., 2002 Mundia et al., 2016; Siever, 2018). In this study, 66% of the participants were between 18 and 35 years old. Age appears to be one of the critical variables as criminal behavior peaks in late adolescence and early adulthood (Shulman et al., 2013), and TBI is also very common in children, adolescents, and young adults (McCormick et al., 2021). Although mild TBI does not result in long-lasting cognitive, physical, or neurological symptoms in most cases (Mosti and Coccaro, 2018), the effects of such injury may be different in the immature compared to the adult brain (Kirkwood et al., 2006). Several other studies of adolescents have shown that head injury is associated with an increase in aggressive behavior (Jones et al., 2018) and other externalizing problems (Anderson et al., 2009; Keightley et al., 2014; Connolly and McCormick, 2019; McCormick et al., 2021).

We found a significant association between TBI and employment, accommodation, and suspension from school (Table 1). Several factors such as emotional support, mental health and participation in work are associated with better quality of life (Steadman-Pare et al., 2001). On the contrary, social isolation and restricted lifestyle coupled with impulsivity may make individuals prone to suicidal behavior and alcohol abuse (Ponsford et al., 2007; Larney et al., 2012). In the tests measuring alcohol use (AUDIT) and suicidal behavior (ideation and attempt), scores were significantly higher in participants with TBI and increased with the frequency of TBIs. Violence related to alcohol abuse has been widely reported, and several factors have been associated with chronic alcohol use and violence, including psychiatric comorbidities such as personality disorders and mood disorders (Siever, 2018; Sontate et al., 2021). Long-term alcohol abuse can also lead to morphological changes in brain areas involved in self-control, decision-making, and emotional processing (Sontate et al., 2021). The correlation found between TBI and suicidal ideation in the offender population is consistent with the literature (Larney et al., 2012).

The association between TBI and criminality is very complex. Study participants who are, perhaps developmentally, impulsive and violent are inherently more risk-taking and less harm avoidant. Thus, pre-existing factors may predispose individuals to TBI and conversely, TBI may contribute to an exacerbation of impulsive behavior and poorer social skills (Williams et al., 2018). The disruption of communication and social learning leads to problematic interpersonal relationships and severe self-identification problems associated with complex trauma (Hart et al., 2012; Williams et al., 2018). Especially in the brains of young people, the neural systems responsible for important socialization skills are still immature and can be more easily disrupted by TBI (Williams et al., 2018). Injury at this stage of life can increase impulsivity, and impair social judgment (Max et al., 2001) and these behavioral changes are likely to increase the risk of offending.

Developing services to reduce the disability associated with TBI and the re-offending risk is a very complex task. We have been impressed by the literature suggesting the high importance of impulsivity and poor self-control as risk factors for offending. Indeed, these considerations, together with the evidence to suggest that SSRIs may reduce impulsivity and aggression prompted us to undertake the ReINVEST study from which the current sample of participants were drawn (Butler et al., 2021). More generally, we hypothesize that targeting adverse neuropsychiatric conditions or syndromes, associated or comorbid with TBI, for which there is an evidence-base supporting efficacy, might yield significant benefits—not only in the quality of life for the patient but in terms of reduced recidivism. Suitable screening for such conditions should be undertaken, allowing for diversion when appropriate. Evidence already exists to support the benefit of antipsychotics for individuals with psychosis and is suggestive of the benefit of stimulants for people with attention deficit hyperactivity disorder (Lichtenstein et al., 2012; Fazel et al., 2014). Both psychosis and ADHD are over-represented in offender populations (Butler et al., 2006; Young et al., 2015). We will soon be reporting our findings from the results of the ReINVEST trial (Butler et al., 2021), examining the possible benefits of SSRIs for impulsive violent individuals who contributed to the present study.

We acknowledge important limitations, several alluded to already. By virtue of the parent study from which these data were drawn, all study participants were violent offenders and all had threshold levels of impulsivity. This selective nature of the sample leads to at least the potential for correlations between some variables to be spurious (Elwert and Winship, 2014). Furthermore, most of the data presented here was based on self-report, with limited available information on the validity and accuracy of individuals’ recall. A previous study (Schofield et al., 2011) suggested that prisoner self-report of TBI is generally accurate when compared with evidence documented in hospital medical records. Subjective reports of a history of TBI may be subject to error and/or recall biases, as can reports on the duration of reported LOC as an index of severity of TBI. There were no control data (i.e., on non-impulsive non-offenders) to use for comparison. Despite these limitations, we considered that, given the unique nature of the sample, the relative richness of the data relating to participant characteristics, including neuropsychiatric phenomena and information on multiple instances of TBI, the narrowly targeted analyses seeking associations between neuropsychiatric phenomena and TBI history could be justified.

In conclusion, the impulsive individuals with histories of violence who participated in this study reported high levels of anger, distress, substance abuse and early life trauma as well as high rates of past TBI and, frequently, multiple TBI with a hard-to-quantify contribution toward their current levels of criminal justice engagement and neuropsychiatric morbidity. Increased public health efforts, targeting social disadvantage and early childhood adversity, are fundamental to minimizing the prevalence of such conditions that present expensive and complex challenges for the criminal justice system, public health and forensic clinicians. Nevertheless, defining the treatable (in adulthood), and treating appropriately, should continue to be rewarding challenges.

## Data availability statement

The original contributions presented in the study are included in the article/supplementary material, further inquiries can be directed to the corresponding author.

## Ethics statement

The study was approved by these independent ethics committees: the University of New South Wales Human Research Ethics Committee (HREC) (HC11390, HC17771), Aboriginal Health and Medical Research Council HREC (822/11) and Corrective Services NSW HREC (09/26576). Approval from the NSW Justice Health and Forensic Mental Health Network HREC (G8/14) will allow participants to continue the study while in detention. The studies were conducted in accordance with the local legislation and institutional requirements. The participants provided their written informed consent to participate in this study.

## Author contributions

VCR and PS defined the objectives for the study. VCR managed the data and analyzed and interpreted the results, and wrote the first draft of the manuscript. PS and TB critically reviewed and edited the final version. BT, LK, RS, PM, JJ, DG, SA, VC, AE, VG, and KW revised the manuscript. All authors read and approved the final manuscript.

## Funding

The ReINVEST trial was initially funded by the National Health and Medical Research Council (GNT0533559) and subsequently by the NSW Department of Communities and Justice. The funding agencies had no role in study design, data collection, data analysis, data interpretation, or report writing.

## Conflict of interest

The authors declare that the research was conducted in the absence of any commercial or financial relationships that could be construed as a potential conflict of interest.

## Publisher’s note

All claims expressed in this article are solely those of the authors and do not necessarily represent those of their affiliated organizations, or those of the publisher, the editors and the reviewers. Any product that may be evaluated in this article, or claim that may be made by its manufacturer, is not guaranteed or endorsed by the publisher.

## Abbreviations

AIAQ: Anger Irritability and Aggression Questionnaire
AUDIT: The Alcohol Use Disorders Identification Test
BDI-II: The Beck Depression Inventory – II
BIS: Barratt Impulsiveness Scale
EIQ: Eysenck Impulsivity Questionnaire
IPDE: The International Personality Disorder Examination
K-10: The Kessler Psychological Distress Scale
LOC: Loss of Consciousness
NSW: New South Wales
OFC: Orbitofrontal cortex
PTSD: Post-traumatic stress disorder
TBI: Traumatic brain injury
SF-12: Short Form-12
STAXI-2: The State Trait Anger Expression Inventory
